# Fracture of the Patella and of the Olecranon

**Published:** 1900-06-23

**Authors:** 


### FRACTURE OF THE PATELLA AND OF THE OLECRANON.

It has long been held that the bad results which sometimes follow fracture of the patella, and also of the olecranon, are due to the stretching of the fibrous material by which in many cases such fractures become united. Of late years, however, it has become pretty obvious that other influences are at work besides mere want of proper apposition and proper union between the fragments, and that among these the most potent is adhesion of the upper fragment to the bone on which it lies—the femur in the one case, and the humerus in the other. Moreover, it has become apparent that in many cases in which use has returned and the material by which the bones had united has seemed to "give way," this has been due not to any defect in the uniting substance, but to the fact that the upper fragment having become firmly fixed to the neighbouring bone the stretching of the uniting material was a necessary preliminary to obtaining of any movement in the joint at all. It, in fact, became apparent that by far the most important point in the treatment of these fractures was to prevent the fixation of the upper fragment. Bony union is a good thing no doubt, and there is an evident loss of mechanical efficiency in a loose and elongated fibrous junction; but no amount of perfection in the union of the fractured surfaces is of much utility, so long as the upper fragment is held fast and blocks the way for the transmission of power from the contracting muscle to the distal portion of the limb. Hence, for some years past, surgeons having seen what are the real obstacles to success in the treatment of the patella or olecranon, have devoted themselves far more than they did in old days to maintaining mobility in the joint, and above all things to preventing that fixation of the upper fragment which is the source and origin of most of the difficulty in restoring the functions of the limb. Speaking on this subject recently at St. George's Hospital, Mr. William Bennett said that in a case of fracture of the patella, in the absence of the treatment by suture now so commonly employed, the final result was dependent mainly upon two points, (1) the amount of laceration of the lateral expansions about the knee at the time of injury (the less these expansions are ruptured the more being the strength left in the joint); and (2) the amount of mobility finally retained by the upper fragment of the patella. If the fragment does not contract inveterate adhesions, and if it finally becomes freely moveable on the femur, a more or less useful limb follows, the strength of the limb as a rule being in inverse proportion to the length of the uniting medium. If, on the other hand, fixation of this upper fragment occurs, then, however good the union, the interference with the mobility and strength of the joint is so great that the patient is greatly crippled. The first object then in the treatment of a case of fracture of the patella, whether it has been wired or not, is to prevent by constant manipulation any chance of adhesion of the upper fragment to the femur—a line of treatment which excludes any form of splint which makes the patella inaccessible. This remark, by the by, is not confined to the treatment of fractures, but applies to all cases of inflammation of the knee joint which are liable to be followed by stiffness, in all which cases fixed, immovable apparatus, such as plaster of Paris, silicate of potash, and the like are rendered entirely inapplicable by the fact that they prevent access to the injured part. The best results after fracture of the patella, when not subjected to the wiring treatment, will, he says, be obtained by immediate smooth massage and patella manipulation, followed in a fortnight or earlier by gentle passive movement of the knee; and it is to be remarked how little, if any, stretching of uniting material occurs in cases treated in this way, if only care be taken from the outset to secure free movement of the patella upon the femur. And what is true of the patella is true also in regard to the olecranon. In cases of fracture of this bone, if the case be treated by prolonged immobility, adhesions take place, and the detached fragment of the olecranon becomes practically fixed to the humerus; perhaps not by bony union, but by such strong adhesions that it is very difficult to move it at all; and since, as with the patella, the utility of the limb depends more upon the mobility of the upper fragment than upon the character or apparent strength of the union, it will be found here also, when the fragment is fixed, that on commencing to bend the joint, movement is obtained not by the gliding downwards of the upper fragment, but by a stretching of the union. The fragment remains unmoved, so that, although flexion may be obtained to a great degree, power of extension—that is, of transmitting motive force from the muscle to the ulna—is almost, if not entirely, wanting. Thus the two great cardinal facts to bear in mind in the rational treatment of fracture of the patella and fracture of the olecranon are, first, the avoidance under all circumstances of any chance of the upper part of the patella or of the olecranon becoming adherent to the subjacent bone, which may be prevented by massage and lateral manipulation, combined with gentle flexion and extension; and second, that all manipulation and passive movement should be preceded by smooth rubbing of the muscles by which spasm is removed.

